# Environmental Factors in Autism

**DOI:** 10.3389/fpsyt.2012.00118

**Published:** 2013-01-18

**Authors:** Andreas M. Grabrucker

**Affiliations:** ^1^WG Molecular Analysis of Synaptopathies, Neurology Department, Neurocenter of Ulm UniversityUlm, Germany

**Keywords:** zinc deficiency, immune system, cytokines, ASD, Shank3, melatonin, risk factor

## Abstract

Autism is a neurodevelopmental disorders characterized by impairments in communication and social behavior, and by repetitive behaviors. Although genetic factors might be largely responsible for the occurrence of autism they cannot fully account for all cases and it is likely that in addition to a certain combination of autism-related genes, specific environmental factors might act as risk factors triggering the development of autism. Thus, the role of environmental factors in autism is an important area of research and recent data will be discussed in this review. Interestingly, the results show that many environmental risk factors are interrelated and their identification and comparison might unveil a common scheme of alterations on a contextual as well as molecular level. For example, both, disruption in the immune system and in zinc homeostasis may affect synaptic transmission in autism. Thus, here, a model is proposed that interconnects the most important and scientifically recognized environmental factors. Moreover, similarities in how these risk factors impact synapse function are discussed and a possible influence on an already well described genetic pathway leading to the development of autism via zinc homeostasis is proposed.

## Introduction

In the last decade, multiple genes have been implicated in autism, collectively accounting for approximately 15% of cases including autism spectrum disorders (ASDs; Abrahams and Geschwind, 2010). In this context, an increasing number of ASDs can be attributed to rare genetic changes that are either inherited or appear *de novo* and further mutations and candidate genes are increasingly identified. Besides a handful of single specific genes that can be associated with autism, the current theory supports the idea of a polygenic inheritance, meaning that multiple genes are likely to be involved that may predispose an individual to develop autism. Although inherited factors might be largely responsible for the occurrence of autism, it is equally clear that genetic aberrations cannot fully account for all cases of autism. The California Autism Twins Study (CATS) for example, with 192 identical and fraternal twin pairs shows a concordance rate of 77% for male monozygotic twins and 50% for female identical twins. The rates among fraternal twins were 31% (male) and 36% (female; Hallmayer et al., 2011). The concordance rate of 31/36% of fraternal twins is higher than the observed rate of 3–14% between siblings of different ages. Thus, in addition to a genetic heritability, common factors such as the shared prenatal environment might play a role in the formation of ASD. Moreover, the increasing prevalence of autism has drawn attention to the potential involvement of toxins in our environment. Extending the theory of pure genetic causes, it seems likely that in addition to a certain combination of autism-related genes, exposure to specific environmental factors might be necessary to trigger the development of autism in some individuals. However, environmental risk factors do not solely cover the exposure to toxins but include all changes other than those on a DNA – level such as maternal nutrition, infection during pregnancy, and prematurity as well as parental age at conception.

This review highlights the role of environmental risk factors in autism in the context of emerging genetic research which suggests that the synapse is an organelle particularly vulnerable to genetic disruption and possibly disruption by related environmental influences. In particular, recent data suggest that immune system abnormalities and altered zinc homeostasis may affect synaptic transmission. Understanding how genetic and environmental risk factors in autism converge at synapses might provide a valuable starting point for future work towards uncovering the patho-mechanisms of autism.

Autism is a developmental disorder and most cases are diagnosed by the age of three and as early as 14 months (Landa, 2008). Nonetheless, autism might be present from birth on rather than develop within this developmental time window. Thus, prenatal environmental factors are of considerable interest for the development of autism (Table 1).

**Table 1:** **Summary of the presented environmental risk factors for autism**.

Risk factor		Reference	Quality of evidence produced by the studies
Prenatal viral infection	Maternal infection, autoimmune disease, and/or allergy could alter the immune status of the fetal brain and the fetal immune system in general	Arndt et al. (2005), Libbey et al. (2005), Miller et al. (2005), Patterson (2009), Pardo et al. (2005), Blattner (1974), Meyer et al. (2007), Fox et al. (2012), Anderson et al. (2007)	The results have been replicated multiple times and the evidence for an association of altered immune status and ASD is strong and growing
Zinc deficiency	A high incidence rate of zinc deficiency is seen in autistic children. Maternal/early developmental zinc deficiency might provide a mechanism of gene/environment interaction	Lakshmi Priya and Geetha (2011), Faber et al. (2009), Jen and Yan (2010), Walsh et al. (2001, 2002), Yasuda et al. (2011), Golub et al. (1995), Sandstead et al. (1978)	The results have been replicated multiple times, recently using a large cohort of 1,967 autistic children. Based on the data, a strong association of zinc deficiency and autism is found
Abnormal melatonin synthesis	Genetic abnormalities and/or environmental factors may influence melatonin synthesis. Melatonin regulates the circadian rhythm, is an antioxidant, is involved in the immune response, and regulates synaptic plasticity	Rossignol and Frye (2011), Cortesi et al. (2010), Melke et al. (2008), Feng et al. (2012)	Few but high-quality studies report an association of abnormal melatonin synthesis and autism. Genetic studies hint towards a decrease in melatonin as causative rather than aftereffect of autism. However, more research is needed to strengthen the association and propose a patho-mechanism
Maternal diabetes	Obesity and diabetes occur more frequently in mothers of ASD cases. Diabetes in the mother during pregnancy leads to a twofold increased risk	Gardener et al. (2009), Krakowiak et al. (2012)	Meta-analysis confirmed maternal diabetes as risk factor. However, the number of studies is small and others have not found a significant association. It is likely that in some cases of diabetes, downstream effects might act as risk factor for autism. However, more molecular biological research is needed to identify the possible patho-mechanisms
Prenatal and perinatal stress	Autism has been reported to be associated with prenatal stress. In animals, regardless of the specific prenatal stressor used, prenatal stress activated the HPA axis resulting in abnormalities in postnatal immune function	Ward (1990), Beversdorf et al. (2005), Kinney et al. (2008), Limperopoulos et al. (2007)	Stress can refer to factors that range from mechanical to purely psychological ones. The best association of “stress” with autism is seen by factors activating the HPA axis, which might be related to alterations in the immune system. Future research will have to closer investigate specific stressors and the related cellular and molecular alteration
Toxins	The incidence of autism is significantly higher in children prenatally exposed to valproic acid or thalidomide. Organophosphate and organochlorine pesticides may contribute to autism as well as psychiatric drugs taken by the mother during pregnancy	Moore et al. (2000), Stromland et al. (1994), Kolozsi et al. (2009), Kumar and Chhibber (2011), Kumar et al. (2010), Karr et al. (2007), Dufault et al. (2012), Roberts et al. (2007), Szpir (2006), Gardener et al. (2009)	A limited number of cases and studies makes the findings hard to interpret resulting in a rather weak association of toxins as risk factor and the development of autism. A more solid association can be found in the use of psychiatric drugs in the mother during pregnancy. However, this association might be explained in a number of ways, which need further investigation
Parental age	The risk to develop autism is associated with advanced age in either parent. While most mutations accumulate in the paternal germline, advanced maternal age might contribute through mechanisms such as increased pregnancy complications and maternal autoimmunity	Gardener et al. (2009), Shelton et al. (2010), Parner et al. (2012), Sandin et al. (2012), Kong et al. (2012), van Balkom et al. (2012), Buizer-Voskamp et al. (2011), Grether et al. (2009), Croen et al. (2007), Reichenberg et al. (2010)	Meta-analysis of multiple studies confirmed parental age as risk factor for ASD. The result is underlined by recent genetic studies specifically revealing an increased paternal mutation rate as possible patho-mechanism
Postnatal risk factors	Gastrointestinal or immune system abnormalities, allergies, and exposure of children to drugs, infection, certain foods or heavy metals have been proposed as risk factor for autism	Iebba et al. (2011), Liu et al. (2005), Sahley and Panksepp (1987), Wills et al. (2007), Cohen et al. (1982), Rossi-George et al. (2011), Kinnell (1985), Cohen et al. (1976)	The evidence for the discussed postnatal risk factors needs further substantiation. While it seems plausible that some of the factors can affect brain development prenatally, their postnatal mode of action needs further investigation

*Summary statements about each of the environmental factors. The quality of evidence produced by the published studies is weighted based on the replicability and strength of association*.

### Prenatal viral infection

Failures in early fetal brain development have been linked to a higher risk for autism and attention has been drawn to offspring exposed to viral or bacterial infections *in utero* (Arndt et al., 2005; Libbey et al., 2005; Miller et al., 2005; Patterson, 2009). For instance, infections that have been associated with autism include prenatal influenza, rubella, and cytomegalovirus infections (Pardo et al., 2005). However, the outcome of exposure to a prenatal viral infection depends on many factors such as maternal immune status, susceptibility of the maternal and fetal host, the developmental stage of the fetus, the amount of virus reaching the fetus, the route of access and the infecting virus, and strain of virus (Blattner, 1974). Nevertheless, given the variety of viruses and their pathogenic effects that can be associated with autism, immunological imbalance in general might be an underlying risk factor for autism (Pardo et al., 2005). This hypothesis is underlined by many reports of abnormalities in peripheral immune cells, as well as associations between variants of genes for cytokines, their receptors, and human leukocyte antigens (HLA) in autism (Patterson, 2009). It was shown that immune changes early in development persist throughout development and aging and are still detectable in the adult brain (Patterson, 2009). Despite these long lasting changes, specific gestational windows may be associated with a higher susceptibility to infection-mediated deregulations in brain development (Meyer et al., 2007).

The risk for autism associated with prenatal infections is most likely dependent on the individual immune status of the mother and fetus. This is substantiated by the finding of a possible association of autism with autoimmune disease and allergy, particularly in the mother (Fox et al., 2012). Consequentially, maternal infection could alter the immune status of the fetal brain and the fetal immune system in general, since the placenta serves as the source of hematopoietic stem cells for the fetus (Gekas et al., 2005). A pathology of the placenta has been linked to the development of autism (Anderson et al., 2007).

### Zinc deficiency

Metal ion homeostasis is essential for proper brain function and its disruption is implicated in severe neurological symptoms and cognitive diseases. Zinc (Zn^2+^) -deficiency has many different effects on the brain. Zn^2+^ is the second most abundant trace element in the body and thus not surprisingly, plays a role in many different processes, such as cell division and differentiation. For instance, hundreds of enzymes require Zn^2+^ for their functions (Takeda, 2001). Many studies have shown that Zn^2+^-deficiency affects several brain functions and interferes with neuronal maturation via impairment of Zn^2+^ dependent systems during early development, resulting in severe brain dysfunctions such as irrecoverable impairment of learning and memory. Approximately 90% of the total brain Zn^2+^ is bound to proteins with free Zn^2+^ mostly located in presynaptic vesicles (Frederickson, 1989). However, transgenic animals without presynaptic Zn^2+^ were not shown to develop symptoms of autism. Recent studies have shown that Zn^2+^ ions are able to modulate the PSD scaffold of synapses via the autism-associated proteins Shank2 and Shank3 (Jan et al., 2002; Baron et al., 2006).

Within the body, copper (Cu^2+^) and Zn^2+^ have competing roles in a way that Cu^2+^ overload leads to Zn^2+^-deficiency (Hall et al., 1979; Huster, 2010). Former studies describe a significant elevation of Cu^2+^ in the hair and nail samples of subjects with autism (Lakshmi Priya and Geetha, 2011). Moreover, further studies indicate that the Cu^2+^/Zn^2+^ ratio is increased in the serum of subjects with autism (Faber et al., 2009) and was suggested as biomarker for children with autism. However, Zn^2+^ deficiency might not only be a biomarker for autism, but indeed a risk factor. Many autistic children have Zn^2+^-deficiency (Walsh et al., 2001, 2002; Jen and Yan, 2010; Yasuda et al., 2011). In a recent study, hair Zn^2+^ concentrations from 1,967 children with autism were investigated and an incidence rate of Zn^2+^-deficiency in the infant-group aged 0–3-year-old was estimated 43.5% in male and 52.5% in female. These findings suggest that infantile Zn^2+^-deficiency may contribute to the pathogenesis of autism (Yasuda et al., 2011).

Zn^2+^-deficiency causes neuropsychological symptoms, learning, and memory impairments (Golub et al., 1995), behavioral problems and an enhancement of glutamate excitotoxicity. Therefore, Zn^2+^-deficiency is also associated with the occurrence of seizures (Grabrucker et al., 2011a) and many patients with autism suffer from epilepsy.

Some candidate genes that have been identified so far to be associated with the development of autism, such as COMMD1 (COMM domain-containing protein 1), MTF1 (metal regulatory transcription factor 1), metallothioneins (MTs), ZnT5 (zinc transporter 5), ERK1 (extracellular signal-regulated kinase 1), TrkB (tyrosine-related kinase B), SHANK2, and SHANK3 (SH3 and multiple ankyrin repeat domains 2/3, also known as proline-rich synapse-associated protein (ProSAP1 and ProSAP2; Serajee et al., 2004; Huang et al., 2008; Grabrucker et al., 2011b; Levy et al., 2011; O’Roak et al., 2011; Nuttall and Oteiza, 2012; Sanders et al., 2012), are influenced by Zn^2+^ or themselves involved in Zn^2+^-signaling and metal ion homeostasis. Thus, Zn^2+^ might provide a mechanism of gene/environment interaction and maternal Zn^2+^-deficiency might act as risk factor for autism. Zn^2+^-deficiency in pregnant rhesus monkeys has effects on the behavior of infants (Sandstead et al., 1978). Intriguingly Zn^2+^-deficiency is known to compromise the immune system, providing a possible link to prenatal infections.

### Abnormal melatonin synthesis

It was hypothesized that abnormalities in melatonin secretion may play a role in the development of autism. Melatonin is an endogenous neurohormone produced in the dark predominantly in the pineal gland and levels of melatonin or melatonin derivatives were found to be often below average in individuals with ASD (Rossignol and Frye, 2011). Along with the decreased levels of melatonin, children with ASD have a higher prevalence of sleep abnormalities such as longer “sleep onset latency,” frequent night-time awakenings and reduced sleep duration (Rossignol and Frye, 2011). Sleep problems in ASD might occur as a result of complex interactions between genetic and social/environmental factors (Cortesi et al., 2010). Circadian abnormalities in autism might be the result of genetic abnormalities related to melatonin synthesis. Deletions of the ASMT (acetylserotonin *O*-methyltransferase) gene, encoding the last enzyme of melatonin synthesis, have been found in several individuals with ASD (Melke et al., 2008). Additionally, environmental factors can influence melatonin synthesis. For instance, maternal stress was shown to result in reduced melatonin levels (Feng et al., 2012) as well as light pollution (Reiter et al., 2006; Falchi et al., 2011). Interestingly, Zn^2+^-deficiency has an effect on various hormones such as melatonin. A reduction in Zn^2+^ levels in pinealectomized rats suggests a reciprocal affect between Zn^2+^ and melatonin and a significant decrease in melatonin levels was observed in Zn^2+^-deficient animals (Bediz et al., 2003).

Although melatonin is best known for its role as a key regulator of the circadian rhythm, it is also a potent antioxidant, has anti-inflammatory properties, is involved in the immune response, and helps regulate synaptic plasticity (Rossignol and Frye, 2011). Given that a low melatonin level can also be found in healthy individuals, it cannot be considered as a direct cause of ASD, but as a susceptibility factor. The effects of maternal melatonin deficiency on the offspring have not been investigated thoroughly in terms of an association with ASDs.

### Maternal diabetes

Meta-analysis revealed diabetes in the mother during pregnancy as a risk factor for autism (Gardener et al., 2009) with a twofold increased risk. Still, the pathological mechanisms remain unknown. In line with these data, a study (Krakowiak et al., 2012) found that metabolic conditions such as obesity, hypertension, or diabetes could occur more frequently in mothers of ASD cases. However, obesity alone was significantly associated with this risk in this study, while only a trend was observed for hypertension and diabetes.

### Prenatal and perinatal stress

Several lines of evidence show significant effects of prenatal stress activating the hypothalamic-pituitary-adrenal (HPA) axis on postnatal behavior in human and animal studies. If autism can result from prenatal stress is currently investigated and the duration, severity, and type of stress are under debate. So far, autism has been reported to be associated with prenatal stress both with retrospective studies (Ward, 1990; Beversdorf et al., 2005) and by comparison to historical events such as storm catastrophes. Moreover, animal studies have reported that prenatal stress produces behaviors resembling symptoms of autism (Kinney et al., 2008). Along with behavioral symptoms significant changes have been found in the regulation of the HPA axis of these animals, regardless of the specific prenatal stressor used. Additionally, prenatal exposure to either stress hormones or psychological stress of the mother in rhesus monkeys resulted in abnormalities in postnatal immune function in infancy that were persistent throughout late childhood (Coe et al., 1999). These immune functions such as proliferation of lymphocytes, natural killer cell activity, and production of cytokines, might decrease the ability to resist viral and bacterial infections and thus the pathological mechanisms of prenatal stress might be related to the increased risk of autism seen with prenatal infections. Stress can also be produced by complications of labor and delivery. Both are associated with greater risk of hypoxia and cerebral hemorrhage in the newborn and a study shows that those who survived cerebellar hemorrhagic injury had a significantly increased risk to develop autism (Limperopoulos et al., 2007).

### Toxins

Independent from maternal health, environmental agents are threatening the development of a fetus, causing birth defects. These so called teratogens have been suggested as potential risk factor for autism. For example, the incidence of autism is significantly higher in children prenatally exposed to the anticonvulsant agent valproic acid (Moore et al., 2000) or the immunomodulatory agent thalidomide (Stromland et al., 1994) early in the first trimester of gestation. Valproic acid for instance, an anticonvulsant used in the treatment of epilepsy and also bipolar disorder due to its mood-modifying ability, is a histone deacetylase inhibitor and might change the methylation pattern of autism – associated genes, thereby affecting their expression rate. Intriguingly, administration of valproic acid *in utero* leads to developmental delays and lifelong deficits in motor performance, social behavior, and anxiety – like behavior in the offspring (Kolozsi et al., 2009). Along with this, Neuroligin 3 (NLGN3) mRNA expression was significantly decreased in hippocampal CA1 region, dentate gyrus, and somatosensory cortex of these animals (Kolozsi et al., 2009). Nlgn3, a member of neuroligin family, is expressed in the postsynaptic compartment of neurons and mediates transsynaptic signaling by interacting with neurexin (NRXN; Südhof, 2008). Mutations in NLGN3 and NRXN have been reported as genetic cause for ASD (Jamain et al., 2003).

Thalidomide, which was infamous for causing birth defects when used as an antiemetic in pregnancy in 1950/1960 still is of pharmacological interest given its immunomodulatory effects (Kumar and Chhibber, 2011). The observed immunomodulation by thalidomide might be related to the increased risk observed through prenatal infection. Thalidomide, for instance, modulates cytokine levels and macrophage pro-inflammatory function (Kumar et al., 2010). It was also found to have anti-angiogenic and immunomodulatory properties including T cell co-stimulation, and activation of NK (natural killer) cells (Quach et al., 2010).

Prenatal exposure to organophosphate pesticides such as diazinon and chlorpyrifos, agents that have been shown to be neurotoxic (Karr et al., 2007), may contribute to autism. A genetic predisposition seems to enhance vulnerability for these substances (Dufault et al., 2012). Additionally, a study found that women who are in the first 8 weeks of pregnancy in closer contact with the organochlorine pesticides dicofol and endosulfan due to their residential proximity to sprayed fields, are several times more likely to give birth to children with autism (Roberts et al., 2007). However, a limited number of cases and studies makes these findings hard to interpret and the concordance rate is not 100%, which suggests that a genetic predisposition might be necessary for a toxin to act as trigger (Szpir, 2006).

A more solid association for a factor for the development of autism can be found in the use of psychiatric drugs in the mother during pregnancy (Gardener et al., 2009). The association of psychoactive medications could be achieved through various ways. Since most of these drugs are able to cross the placenta, the effect might indeed be caused by direct exposure to the psychoactive substance. Nevertheless, the actually treated condition of the mother might itself have an influence on fetal development or reflect certain genetic traits that are possibly shared between autism and the mother’s condition. Moreover, the need of psychoactive medication in the mother may hint towards a prenatal stress situation, a condition that has been linked to autism before.

### Parental age

The risk to develop autism is associated with advanced age in either parent (Gardener et al., 2009; Shelton et al., 2010; Parner et al., 2012). A meta-analysis of epidemiological studies including 25,687 ASD cases and 8,655,576 control subjects investigating the association between maternal age and autism supports advanced maternal age as a risk factor for autism. The association still persisted after considering effects of paternal age (Sandin et al., 2012). Further studies indicate that the father’s age is of similar importance given that men transmit a much higher number of mutations to their children than women (Kong et al., 2012) and that the age of the father is the dominant factor in determining the number of *de novo* mutations in the child (Kong et al., 2012). Thus, advanced paternal age was similarly associated with increased risk of ASDs in offspring (Croen et al., 2007; Grether et al., 2009; Reichenberg et al., 2010; Shelton et al., 2010; Buizer-Voskamp et al., 2011; van Balkom et al., 2012). However, advanced paternal and maternal age may act as risk factors through different mechanisms. While most small-scale *de novo* mutations accumulate in the paternal germline over time related to parental age (Iossifov et al., 2012), eggs are fully developed at the time of a mother’s birth. Thus, advanced maternal age might contribute as risk factor for autism through mechanisms such as increased pregnancy complications (Gardener et al., 2009) and maternal autoimmunity (Enstrom et al., 2009). The risk factor of maternal autoimmunity again hints towards the involvement of an immune response during pregnancy as a risk factor for autism. Pro-inflammatory cytokines possibly arising from maternal autoimmunity might cause aberrant neuronal growth and function within the developing fetal brain (Buehler, 2011).

### Postnatal risk factors

Although it is assumed that children are born with autism, a wide variety of postnatal factors contributing to autism have been proposed (Table 1). However, the evidence for these risk factors, such as gastrointestinal or immune system abnormalities, allergies, and exposure of children to drugs, infection, certain foods, or heavy metals needs further substantiation. Vaccination can no longer be regarded as risk factor for autism (Doja and Roberts, 2006). Evidence from several rigorous scientific studies examining an association between vaccine use and autism have not identified such a link (Miller and Reynolds, 2009) and the data of former studies claiming an association were shown to be scientifically fraudulent (Flaherty, 2011).

Gastrointestinal abnormalities, in terms of the “Leaky gut syndrome” as risk factor for autism are based on parents reporting gastrointestinal disturbances in autistic children, and several studies have investigated possible associations between autism and the gut (Liu et al., 2005; Iebba et al., 2011). Additionally, a connection between autism and opiates was proposed (Sahley and Panksepp, 1987). Caused by a digestive disorder, gluten, and casein might be converted to the opioid peptides gliadorphin and casomorphin, thus influencing the development of autism. However, more evidence is needed to verify that autistic children are more likely to have gastrointestinal pathologies than control children. Nevertheless, it is tempting to speculate that a possible link between malnutrition, gastrointestinal abnormalities, and Zn^2+^-deficiency reported as risk factors for autism may exist. Similarly, Zn^2+^-deficiency might influence the immune system and it was hypothesized that autoantibodies that compromise brain development or important metabolic pathways may be a risk factor for autism. Similar to maternal antibodies, via an autoimmune mechanism, own antibodies may cause an aberrant immune activity (Wills et al., 2007).

Heavy metal poisoning by mercury and lead have been associated with autism. Given that elevated lead levels in the blood of autistic children have been found, lead poisoning has been suggested as a possible risk factor (Cohen et al., 1982). Lead and stress share biological substrates and produce common adverse effects (Rossi-George et al., 2011). However, the evidence so far is indirect for the association between autism and mercury or lead exposure after birth. Moreover, atypical eating behaviors of autistic children, such as seen in “pica” (the persistent eating of non-nutritive substances for a period of at least 1 month; Kinnell, 1985) may influence lead levels more than being a consequence of autism (Cohen et al., 1976).

## Environmental Factors in Autism – a Model

**Figure 1:**
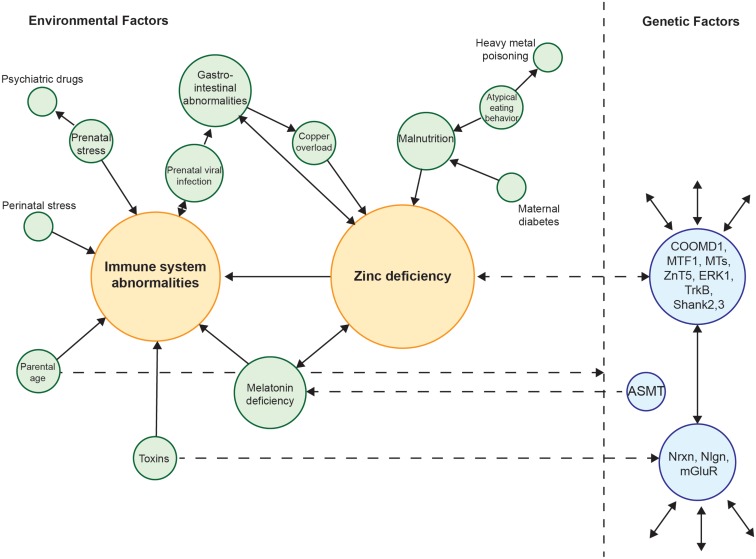
**Interconnection of various environmental risk factors for autism**. Many risk factors for autism can be related to each other. All factors are shown weighted for their interconnectedness with other factors. Zn^2+^-deficiency and immune system aberrations receive most connections and thus can be placed in the center of the network. Zn^2+^-deficiency, toxins, and the parental age may act on genetic factors providing a crosstalk between genes and environment. Zn^2+^-deficiency and melatonin deficiency in turn can also be caused by genetic alterations.

Taken together, there is overwhelming evidence that autism is a genetic disorder. Twin studies indicate a genetic basis for autism and genome wide association studies have revealed some interesting candidate genes (Miles, 2011; Li et al., 2012). Nevertheless, environmental factors can have an obvious influence on the development of autism. It is tempting to speculate that most of the identified risk factors so far, such as prenatal stress, prenatal infection, maternal Zn^2+^-deficiency, and maternal exposure to toxins as well as potential novel factors that will be identified in the future might indeed all be different sides of the same coin. Thus, the search for similarities in all the identified genetic as well as environmental factors might lead to a time window in brain development, where certain brain regions and specific pathways are prone to disruption caused by various agents but all leading to the phenotype of autism. Figure 1 shows an attempt to unite the several environmental risk factors identified in autism so far and tries to bridge the gap between these and genetic factors (Figure 1). From this analysis, two central motifs, immune system abnormalities, and Zn^2+^-deficiency, emerge.

A close connection exists between a functional immune system and the development of the central nervous system and a successful neurodevelopment requires a normal balance of immune responses (Ashwood et al., 2006). Immune system abnormalities might result from toxins that compromise important players of the system, stress, parental age, Zn^2+^-deficiency, and infections.

Prenatal stress in turn was associated with an abnormally developing HPA system, an affected placenta as well as hippocampal volume reduction. Smaller hippocampal volumes in turn are associated with an increased secretion of cortisol (Frodl and O’Keane, 2012). Intriguingly, patients with ASD were characterized by a significant reduction of gray matter volume in the amygdala-hippocampus complex (Via et al., 2011). Cortisol as the primary end product of the HPA axis is an important component of the stress system in humans and essential for fetal growth and the induction of specific enzymes (Charil et al., 2010). Therefore, women have naturally elevated levels of cortisol during pregnancy. However, it is possible that maternal cortisol concentrations can reach abnormally high levels under particular stressful conditions. Cortisol that reaches the fetus may potentially alter fetal development and growth. Animal studies have shown that fetal exposure to high levels of cortisol alters neuronal development and results in a smaller hippocampal size (Charil et al., 2010).

The placenta is similarly affected by prenatal stress and releases Corticotropin releasing factor (CRF) from the placenta during sub-acute and chronic prenatal stress that may cross the blood-brain barrier of the fetus. Consequently, the function and integrity of the hippocampus is affected, for instance by an increase in release of acetylcholine, inhibiting the influence of cholinergic receptors on the activity of the HPA axis (Charil et al., 2010).

Parental age is correlated with an increased risk of maternal autoimmunity as the majority of autoimmune disorders are manifest between 30 years of age and upward (Ashwood et al., 2006). Autoimmune disorders arise when the immune system is inappropriately directed to recognize and response to self-components and maternal autoantibodies may affect fetal brain development during critical periods of neurodevelopment (Chonchaiya et al., 2010). Components of the immune system such as cytokines and autoantibodies, may act to increase fetal cytokine signaling and expression, disrupt trophic factor, and apoptotic signaling, as well as modulate cytoarchitecture in the CNS (Murphy et al., 2010). Prevalence rates suggest that autoimmune diseases, such as rheumatoid arthritis, autoimmune thyroid diseases, myasthenia gravis, multiple sclerosis, systemic lupus erythematosus (SLE), and primary biliary cirrhosis are not uncommon, affecting approximately 5% of women and 3% of men in the USA (Murphy et al., 2010).

Prenatal infections could persistently alter the immune status of the fetus. An inflammatory-like state has been detected in postmortem brains of autism patients, indicated by elevated cytokines and activated microglia and astrocytes. Moreover, cytokine elevation was also found in the cerebrospinal fluid (CSF) of living autistic children (Pardo et al., 2005). Cytokine IL-6 thereby attracted special attention. IL-6 leads to a reduction in insulin-like growth factor (IGFs)-binding protein 3 and IGF1 (IGFs 1; Patterson, 2009). Intriguingly, IL-6 also regulates Zn^2+^ binding MTs (Manso et al., 2011). Animal studies suggest that these inflammatory changes might ultimately alter NMDA receptor mediated excitatory synaptic transmission and plasticity in the hippocampus (Escobar et al., 2011). The inflammatory-like state was also detected in the peripheral immune system. Immune cell may infiltrate into the colon, ileum, and duodenum and an increased T cell activation can be present in the intestinal mucosa. Thus, these inflammatory changes may also lead to gastrointestinal abnormalities. However, in terms of prenatal infections it is unclear, if the infection itself causes an imbalance of the immune system or if a compromised immune system in the first place leads to an increased susceptibility for infections.

The second central environmental factor is Zn^2+^-deficiency. Zinc is an essential trace element and plays various roles in biological processes. For instance, Zn^2+^ is a structural component of proteins and involved in enzymatic processes and a transcriptional regulator (Tuerk and Fazel, 2009). Zn^2+^-deficiency itself influences the immune system and low Zn^2+^ status has been associated with an increased susceptibility for infections (Wong and Ho, 2012). Zn^2+^-deficiency significantly impairs both adaptive and innate immune responses, and promotes systemic inflammation. Zn^2+^ is essential for normal development and function of cell-mediating innate immunity, neutrophils, and natural killer cells. For instance, macrophages, phagocytosis, intracellular killing, and the growth and function of T and B cells are all affected by Zn^2+^-deficiency. Zn^2+^-deficiency also affects the production, secretion, and functions of cytokines (see thalidomide), the basic messengers of the immune system (Prasad, 2009). Cytokines have widespread effects on neuronal pathways and are able to show behavioral effects such as alteration of mood and sleep (Ashwood et al., 2006). Imbalances in the immune system were seen in humans with induced mild Zn^2+^-deficiency within 12 weeks of a Zn^2+^-restricted diet (Prasad, 2009).

Zn^2+^ homeostasis is also highly regulated via the gastrointestinal tract (Tuerk and Fazel, 2009) and gastrointestinal abnormalities have been mentioned as risk factor for autism. Moreover, viral infections might affect the gastrointestinal system providing another link between immune system abnormalities and Zn^2+^-deficiency. However, Zn^2+^-deficiency can be caused by many factors that have been associated with autism in the past, such as maternal diabetes, malnutrition, copper overload, and atypical eating behavior. Maternal diabetes and obesity have been associated with this risk to develop autism and may influence the developing child similar to malnutrition. Vegetarian diet usually provides a low intake of Zn^2+^ (Craig, 2010) and also fiber rich diet, suggested for diabetes (Wolfram and Ismail-Beigi, 2011), increases the risk of Zn^2+^-deficiency (Foster et al., 2012). Intriguingly the consumption of fiber rich diet within the USA increased in the last decades (Hiza and Bente, 2007).

Within the body Cu^2+^ and Zn^2+^ have competing roles in a way, where Cu^2+^ overload leads to Zn^2+^-deficiency (Hall et al., 1979; Huster, 2010). Cu^2+^ overload in an extreme form is seen in patients suffering from Wilson’s disease, where homozygous mutation of the ATP7B gene results in Cu^2+^ accumulation in tissues manifesting in neurological or psychiatric symptoms among others. Although the disease is rather rare, the heterozygous form with only one faulty copy of the affected gene is not so rare. It was speculated that in this case, the developing fetus of a pregnant woman may experience major difficulties in the early development of the brain due to a Cu^2+^ overload – induced Zn^2+^-deficiency, which may later manifest as autism (Johnson, 2001). Atypical eating behaviors in autism, such as pica, in turn may influence again the nutritional status but also cause heavy metal poisoning. In this case, both scenarios would be postnatally affecting the development of autism. Nevertheless, a heavy metal intoxication during pregnancy might again disturb Zn^2+^ homeostasis.

Melatonin deficiency may be the result and cause of a Zn^2+^-deficiency. Melatonin also has an impact on the immune system, thus possibly influencing both major identified environmental factors for the development of autism.

Severe Zn^2+^-deficiency is rare and mostly occurs in developing countries. Marginal Zn^2+^-deficiency in contrast, is a potentially widespread problem. Based on a study of 14,770 individuals aged 3–74 years, the prevalence of Zn^2+^-deficiency in the USA was estimated with 1–3 and 10% of the US population consumes less than half the recommended level for Zn^2+^ and thus is at risk for Zn^2+^-deficiency (Tuerk and Fazel, 2009; Wong and Ho, 2012). Moreover, women may not ingest adequate amounts of Zn^2+^ during pregnancy (Halas and Sandstead, 1975) and suffer from decreased levels of Zn^2+^ during the latter part of pregnancy (Halsted and Smith, 1970; Sandstead, 1973). However, since Zn^2+^-deficiencies can be found in many brain disorders such as schizophrenia and depression (Grabrucker et al., 2011a), more research is needed to investigate the cause of these Zn^2+^-deficiencies.

Intriguingly, environmental factors such as Zn^2+^-deficiency might have a crosstalk with genetic factors. Some autism candidate genes that have been identified so far, such as COMMD1, MTF1, MTs, ZnT5, ERK1, TrkB, Shank2, and Shank3 (Serajee et al., 2004; Huang et al., 2008; Grabrucker et al., 2011b; Levy et al., 2011; O’Roak et al., 2011; Nuttall and Oteiza, 2012; Sanders et al., 2012), are influenced by Zn^2+^ or themselves involved in Zn^2+^ signaling and metal ion homeostasis. The idea that many genes implicated in autism might converge on a single pathway present at excitatory glutamatergic synapses has recently been raised by multiple studies and a Nrxn-Nlgn-Shank pathway has been proposed (Bourgeron, 2009). Thus, Shank proteins, via their dependency on Zn^2+^ ions (Grabrucker et al., 2011c), might influence further autism-associated proteins. For instance, mGluR5, an important target in fragile-X-syndrome is an interaction partner of Shank proteins (Lim et al., 1999). However, it is possible that other identified autism-associated genes have different crossing points with the environmental risk factors for autism discussed here and future research will picture the model of environmental factors influencing genetic predispositions for autism in more detail.

## Environmental Factors in Autism – Modes of Action

Given this interconnectedness of many environmental and genetic factors associated with the development of autism, one can speculate how these risk factors might converge an a single hypothetical pathway by looking at possible similarities in the mode of action. Autism is caused by abnormalities in the structure or functions of the brain, but the underlying biological patho-mechanisms are currently not well understood. However, various recent studies have identified copy number variations (CNVs) as well as single nucleotide polymorphisms (SNPs) of neuronal genes in the genomes of ASD patients. Many of these genes encode proteins that are crucial components of excitatory glutamatergic synapses, including scaffold proteins such as Shank2, Shank3, SAPAP2 from the GKAP/SAPAP family of Shank interacting proteins, neural adhesion molecules, such as Neuroligins, Neurexins as well as Cadherins, Contactin, Contactin-associated protein, and other cell-adhesion molecules (CAMs), calcium channels, and neurotransmitter receptors (Betancur et al., 2009; Pinto et al., 2010; Toro et al., 2010). Moreover, protein kinases and proteins that regulate protein syntheses have been identified as candidate genes associated with ASD. Mouse models for these genes underline their role in autism and hint towards a deregulation of excitatory synapses since all these proteins play a role at the synapse of glutamatergic neurons. Thus, these data imply a link between a synaptic pathology and the characteristics of ASD and shift the synaptic compartment in the focus of interest.

**Figure 2:**
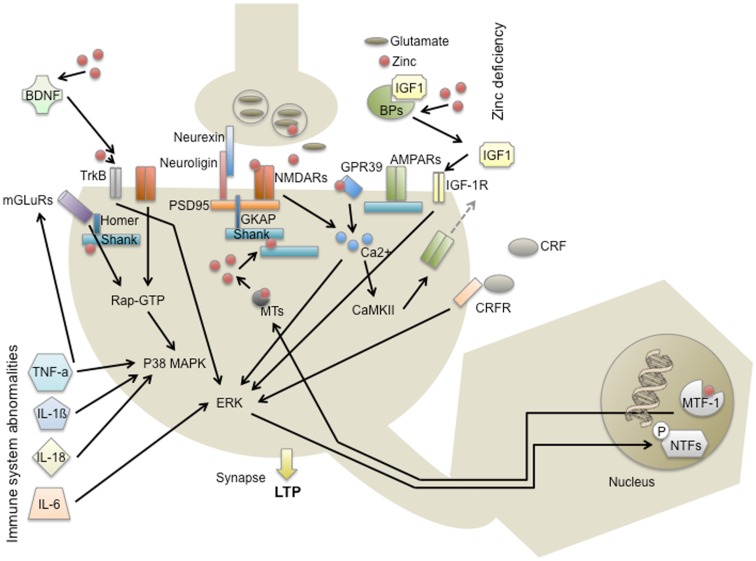
**Modes of action of environmental risk factors for autism at excitatory synapses in the CNS**. Scaffold proteins within the PSD such as proteins of the Shank family build a dense meshwork of interacting protein complexes including Homer, mGluR, AMPAR, and GKAP further connecting to PSD95 and NMDAR. These synaptic components are regulated via signaling cascades such as the p38 MAPK and ERK pathway and altogether ultimately determine the immediate and long-term response to a synaptic stimulus. Environmental factors that have been discussed in autism may influence this signaling outcome on various levels. Zn^2+^-deficiency for instance might be able to alter the PSD scaffold via Shank2 and Shank3 and additionally influences NMDAR, TrkB receptor, and GPR39 receptor signaling. Moreover, Zn^2+^ that might be released from presynaptic vesicles or postsynaptic metallothioneins can affect IGF1 signaling and gene expression via MTF1. Immune system abnormalities, including the expression of cytokines such as TNF-α, Il-1β, IL-18, and IL-6 will affect mGluR, p38 MPAK, and ERK signaling, pathways that are also activated by the receptors mentioned above.

To study a crossing point of environmental factors with genetic predispositions and the development of autism, excitatory synapses may provide a useful starting point. Given that a synaptopathy is the underlying mechanism for autism and focusing on the two central environmental factors “immune system abnormalities” and “zinc deficiency,” similarities can be found influencing synaptic function and plasticity (Figure 2).

Abnormalities in the immune system are known to result in altered cytokine and major histocompatibility complex (MHC) expression and release of nitric oxide (NO) and cyclo-oxygenase products on a molecular level. Thus, inflammatory responses can have several effects on the brain. Circulating immune cells may infiltrate the CNS, resident cells are activated and pro-inflammatory mediators such as cytokines are produced and released. These immune diffusible mediators may influence synaptic processes such as long-term potentiation (LTP; Di Filippo et al., 2008), a molecular mechanism thought to underlie synaptic plasticity in learning and memory formation. LTP manifests as a persistent increase of the evoked response following repeated synaptic activation. The generation of LTP requires activation of postsynaptic NMDA receptors, subsequent postsynaptic Ca^2+^ entry together with the induction of several signaling systems and finally gene transcription and new protein synthesis (Malenka and Nicoll, 1999). Cytokines and other molecules overexpressed during the neuroinflammatory process act as neuromodulators with pro-inflammatory cytokines showing a detrimental effect on neuronal function, viability, and plasticity (Allan and Rothwell, 2001) even when expressed at low levels. In particular, the release of neurotransmitter is influenced as well as signal transduction mechanisms within the postsynaptic density (PSD) and, finally, the ability to induce LTP.

For instance, IL-1 crucially influences several key molecular components of LTP – induction, such as NMDA and AMPA receptor signaling (Coogan and O’Connor, 1997; Lai et al., 2006) and glutamate release (Murray et al., 1997). Similarly, IL-18, a member of the IL-1 family, is able to impair the induction of LTP, probably by negatively modulating NMDA receptor mediated transmission in the hippocampus (Curran and O’Connor, 2001). However, the effects of IL-1 on NMDA and AMPA receptors have not been completely elucidated to date and might be brain region specific. IL-6 is thought to modulate the ERK pathway, a signaling pathway involved in the generation of LTP. Additionally, an increase in IL-6 leads to a reduction in IGFs-binding protein 3 and IGF1 (Patterson, 2009). Intriguingly, IL-6 also influences one of the major Zn^2+^ stores in postsynapses, the Zn^2+^ binding MT proteins (Manso et al., 2011).

IL-18, IL-1β, and TNF-α (Tumor necrosis factor alpha) act on AMPA and NMDA as well as metabotropic receptor signaling, thereby modulating the activity of the stress activated p38 MAPK. TNF-α, a pro-inflammatory cytokine binds to receptors, which are expressed on neurons and glial cells throughout the CNS. Interestingly, the inhibition of mGluR1 and mGluR5 can block the TNF-α – dependent inhibition of early and late LTP. Thus, a combined activation of TNF receptors and mGluRs may impair the formation of LTP, although the exact mechanism of TNF and mGlu receptor activation is still not well understood (Pickering et al., 2005).

Besides their role as modulators of brain growth, IGFs also influence brain plasticity. Specifically, IGFs regulate synapse formation, neurotransmitter release, and neuronal excitability via posttranslational modification of NMDA and AMPA receptors (Torres-Aleman, 1999). IGF1 acts primarily on the IGF1 receptor, which is neuronally expressed and coupled to the PI3K-AKT signal transduction pathway that positively influences cellular growth and thus affects the maturation of outgrowing neurites and synapse like structures. In the case of IGF1, this feature has already been used therapeutically in preclinical settings, where the application of recombinant IGF1 was demonstrated to improve synaptic maturation in a mouse model for Rett syndrome, a type of ASD (Tropea et al., 2009). Moreover, a crosstalk between IGFR activation and the ERK pathway can be found.

Thus, immune system abnormalities might ultimately alter NMDA receptor mediated excitatory synaptic transmission and plasticity (Escobar et al., 2011). Similar results have already been shown in animal studies where changes in NMDAR responses and NMDAR dependent LTP as well as an imbalance in NMDAR levels and subunit composition were reported in recent mouse models for autism (Bangash et al., 2011; Wang et al., 2011; Schmeisser et al., 2012; Won et al., 2012).

Prenatal stress might additionally contribute to the above – mentioned effects via CRF secretion. Stress in known to influence synaptic transmission in brain regions, such as hippocampus, ultimately affecting learning, and memory. Whereas physiological levels of CRF augment memory processes, exposure of high levels of the peptide results in retraction of dendritic spines of excitatory synapses in the hippocampus and amygdala and thus to synapse loss. Although only a minority of dendritic spines is lost due to long lasting CRF exposure, a profound memory impairment and loss of LTP can be observed (Chen et al., 2012). However, the molecular mechanisms of this synapse loss are not fully understood. Intriguingly, CRF activates, through CRF1, the ERK pathway in the area CA3 of the hippocampus.

The idea of Zn^2+^ being a regulatory core component within PSDs results in novel insights and understanding concerning synapse formation and might hint towards a mechanism of Zn^2+^-deficiency in autistic patients. Indeed several autism-associated genes that can be found at excitatory postsynapses, such as Shank2 and Shank3, MTs and ERK are Zn^2+^ binding, and Zn^2+^ regulated proteins. Shank proteins further connect to mGluR, GKAP, Neuroligins, and Neurexins. To date, the source of postsynaptic Zn^2+^ involved in synaptic plasticity is still unclear and might include presynaptic vesicular Zn^2+^ and/or postsynaptic activity dependent release from Zn^2+^ stores such as MTs. Focusing on excitatory synapses, it has been shown that Zn^2+^ influences the activity of glutamate receptors. NMDA receptors have a very high sensitivity to extracellular Zn^2+^ and the activity of GluN2A subunit containing NMDA receptors is inhibited allosterically at low nanomolar concentrations, while at micromolar Zn^2+^ concentrations that may occur during synaptic activity, Zn^2+^ binds to the GluN2B subunit, inhibiting GluN2B-containing receptors (Sensi et al., 2011).

Zn^2+^ also activates a specific metabotropic Zn^2+^-sensing receptor GPR39, which triggers IP3 production, followed by intracellular Ca^2+^ and subsequent activation of ERK and CAMKII-dependent pathways (Besser et al., 2009). Moreover, Zn^2+^ directly promotes the transactivation of the BDNF (brain-derived neurotrophic factor)-related TrkB pathway (Huang et al., 2008) and maternal Zn^2+^-deficiency decreases BDNF expression in the offspring (Chowanadisai et al., 2005). In a recent study, NTFs (neurotrophic factors) were measured in 414 ASD cases and 820 controls. ASD cases were more likely to have significantly decreased BDNF levels and significantly lower TGF-β (transforming growth factor beta) levels were detected in females with ASD. These data hint towards decreased NTFs levels during neonatal period as risk factor for ASD contributing to the pathophysiology of ASD through impairments of neuroplasticity (Abdallah et al., 2012).

Given that Zn^2+^ increases IGF1 receptor sensitivity, Zn^2+^ also affects IGF1 signaling in a way that Zn^2+^-deficiency may decrease IGF1 receptor binding (McCusker and Novakofski, 2004). In general, neurons control cytosolic Zn^2+^ levels tightly using several ZnT transporters, ZIP transporters, and Zn^2+^ buffering MTs and Zn^2+^-deficiency might influence all of the above – mentioned pathways and proteins. Thus, the many faces of Zn^2+^ taking part in such a high number of biological and synaptic processes makes it hard to pinpoint the mechanism involved in the development of autism and the presented model might have to be expanded in the future.

Taken together, two major targets of environmental factors contributing to the development of autism can be found on a synaptic level. The first is the structural composition of synapses that might be altered through deletions or mutations in key scaffold or CAMs such as Neurexin, Neuroligins, GKAP, and proteins of the ProSAP/Shank family. This might ultimately result in changes in receptor number, or composition influencing NMDAR, AMPAR, and mGluR and affect signaling components within the PSD. For instance, Zn^2+^ depletion was shown to cause a decrease in Shank3 and NMDAR levels of forming synapses *in vitro* (Grabrucker et al., 2011c). Loss of Shank3 in turn affects transsynaptic signaling via Neuroligin-Neurexin complexes (Arons et al., 2012) and mGluR5 signaling (Verpelli et al., 2011) and Shank3 knock-out mice display a reduction of AMPAR and NMDAR (Bozdagi et al., 2010; Bangash et al., 2011). Intriguingly, prenatal exposure to valproic acid leads to reduced expression of the synaptic adhesion molecule Neuroligin 3 in mice (Kolozsi et al., 2009).

The second major target is the p38 MAPK (mitogen-activated protein kinase) and ERK pathway. ERK regulates two cellular processes closely associated with plasticity: synaptic delivery of AMPAR (Zhu et al., 2002), and activity dependent dendritic spine modifications (Goldin and Segal, 2003). ERK kinases are themselves heavily influenced by the local Zn^2+^ concentration (Nuttall and Oteiza, 2012). Additionally, ERK2 knock-out mice exhibit marked anomalies in multiple aspects of social behaviors related to facets of ASDs and impaired long-term memory (Satoh et al., 2011). Moreover, Zn^2+^ signaling via GPR39 further alters the receptor composition of excitatory synapses. Thus, both, alterations in synapse morphology induced by structural changes and disturbed signaling by kinases might converge leading to disrupted LTP formation and plasticity at synapses. Therefore, a specific decrement in excitatory signaling caused by various factors, such as genetic mutations or environmental factors like immune system abnormalities or Zn^2+^-deficiency, will ultimately lead to an imbalance of inhibition and excitation. An imbalance of excitation and inhibition was already proposed as underlying cause of autism (Rubenstein and Merzenich, 2003).

Of course, the proposed model is an oversimplification and should be considered a starting point rather than a finalized concept. Many studies have not been considered here and might add, revise, or refine the interactions described above. For instance, the incorporation of novel risk factors or brain region specific effects might deepen the understanding of environmental factors in autism and of autism itself.

## Conclusion

Experimental research often is designed in a way that one component of a system is manipulated and the various outcomes documented. For example, a transgenic mouse model with the knock-out of a specific gene might display a pleiotropy of symptoms depending on the specific role of the encoded protein. However, the cause for these various symptoms is known, since only a single gene has been manipulated.

In the case of autism, current research faces the inverse situation. Many symptoms are observed and many significant associations of environmental as well as genetic factors were found. What is missing so far is a hypothesis, unifying all those different factors chasing autism back to a single cause, for instance the disturbance of a specific synaptic pathway that is influenced by genetic and environmental factors. The fact that many autism-associated genes are interconnected and that also many environmental factors seem to be related and impact genetic factors makes this goal look achievable in the future. Like a necklace of pearls, where a cut between two single parts at a random position in the chain will lead to the drop of every pearl, the deregulation of this environmentally influenced genetic pathway at any level may results in autism. However, it is likely that the cut in the signaling chain might influence the occurrence and severity of specific features of autism.

## Conflict of Interest Statement

The author declares that the research was conducted in the absence of any commercial or financial relationships that could be construed as a potential conflict of interest.
